# Supplementary material for: Linkage Between the Environment and Individual Resilience to Urban Flooding: A Case Study of Shenzhen, China
Source: Int J Environ Res Public Health. 2019 Jul 18;16(14):2559. doi: 10.3390/ijerph16142559 (PMC6678611; doi:10.3390/ijerph16142559)
Supplement: Supplementary file 1 [file ijerph-16-02559-s001.pdf]

Supplementary Information:

Drainage capacity Calculation

Drainage capacity in this research is evaluated based on the model of SWMM and the steps for building SWMM are described in the following sections.

(1) Sewerage System Generalization of Study Area

The process of water accumulation and drainage is complicated in urban areas. The hydrological properties are quite different for permeable and impermeable surfaces. Even for permeable surfaces, different land use types show different properties. The diverse and intricate surfaces with uneven properties in urban areas make it difficult to perform realistic simulations. The sewerage systems of big cities have complicated and fragmented distribution systems, which further increase the difficulty to simulate the accumulation and drainage processes of rainwater. Therefore, it is necessary to simplify and generalize the land use and the sewerage system of the study area.

Underlying Surface Generalization

The study area covers 37.68 km^2 and has different landscape types. Using the land use map and the hydrographic features, the study area can be divided into grass, forest, farmland, bare lands, gardens and green lands, vacant lands, water areas, low-density construction lands (industrial lands and public facilities lands), high-density construction lands (urban construction lands and wholesale and retail lands), and transportation lands. The generalized land use map is shown as Figure S1.

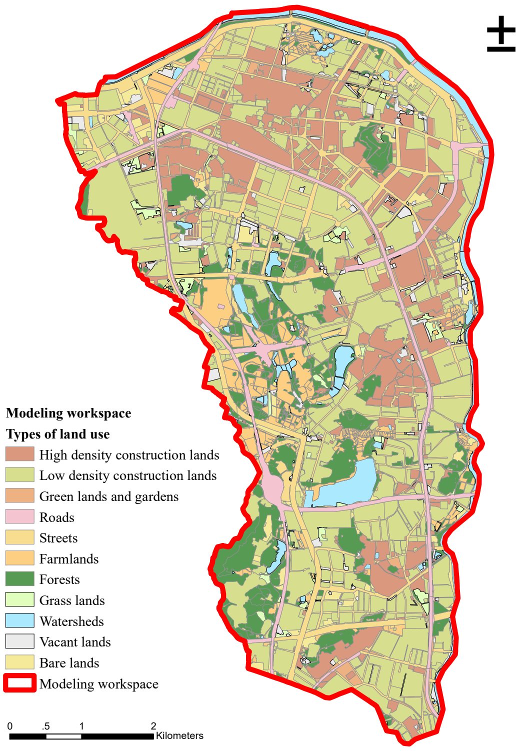

Figure S1. Generalization map of land use.

Pipe and Pipe Junction Generalization

The number of sewerage pipes and pipe junctions is too large for the model therefore they should be simplified for the simulation. The rules for pipe and junction generalization are as follows:

a. The minimum distance between the two junctions is 100 m,

b. Keep the corner junctions, changing diameter junctions, or large variation range of slope junctions,

c. Keep the parallel pipes and junctions on both sides of the roads,

d. Delete the spur pipelines of roads and try to keep the drainage capacity of pipelines the same during the generalization.

Original distributions of pipelines and junctions have two sources; one is from the land use map of Shenzhen (shp) and the other is from sewerage system project map (dwg). Based on the rules and the original base maps, all the pipelines and junctions of study area were simplified to 1151 junctions, including 56 outlets, 1095 inspection wells, and 1044 pipelines, including 49 gouges. Based on the slope and depth of the pipeline junctions, we calculated the flow directions of different pipelines, and the coordinates and length of the pipelines using the module of Calculate Geometry of ArcGIS10.2. Finally, the spatial distribution of pipelines and pipe junctions was simplified as shown in Figures S2 and S3.

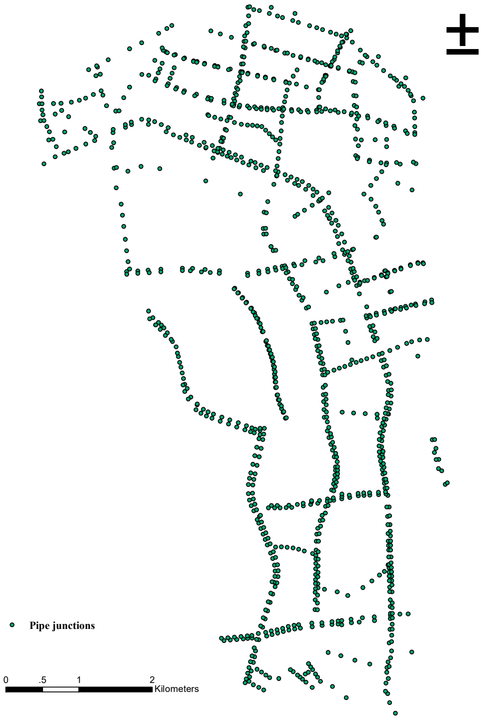

Figure S2. Generalization of pipe junction.

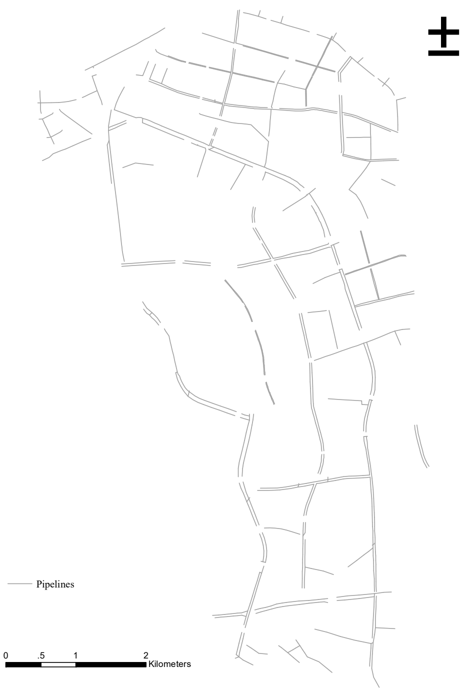

Figure S3. Generalization of pipelines.

Catchment Partition

DEM (Figure S4) is the original data to determine the catchment partition. Using the module of Spatial Analyst Tools (Surface) of ArcGIS 10.2, DEM was completed, slope and flow direction were calculated, and the flow path of water, river networks, and the basins of the study area were obtained. Finally, the basin partition was calculated.

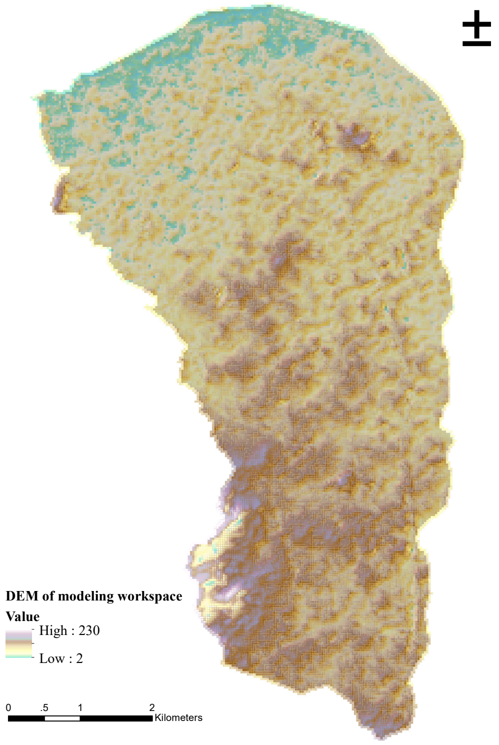

Figure S4. DEM of modeling workspace.

The extracted basins cannot exactly reflect the properties of the underlying surfaces of the study area because the precision of DEM is limited. Since most of the study area includes developed land rather than natural lands, the basin partition process based only on the DEM is not very accurate. Therefore, it is necessary to perform further manual repartition by adding the file of the statutory plans of the study area to ArcGIS 10.2 and combining the partition results of basins with the repartition of the catchments of the study area. Finally, considering the land use types, zoning distributions, and outfalls of the junction distributions, the study area was divided into 1116 catchments (Figure S5).

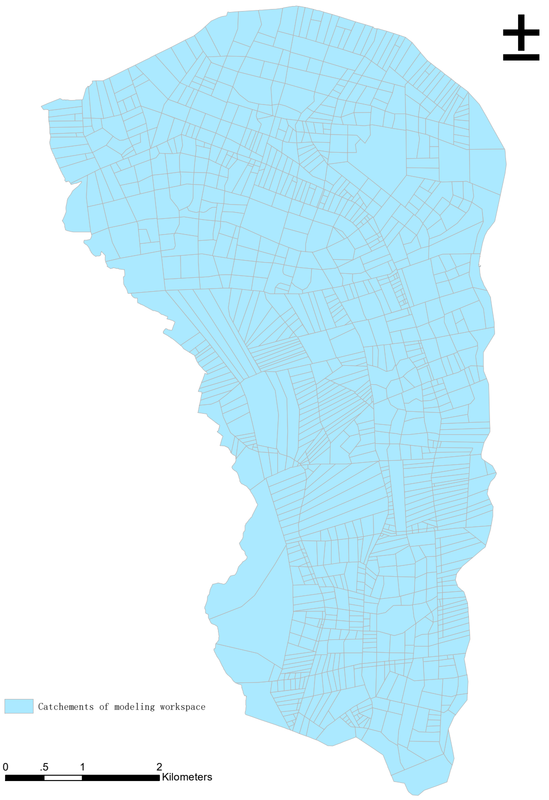

Figure S5. Catchments of modeling workspace.

The catchments, pipelines, pipe junctions were added into SWMM with the spatial coordinates and the study area in SWMM are shown in Figure S6.

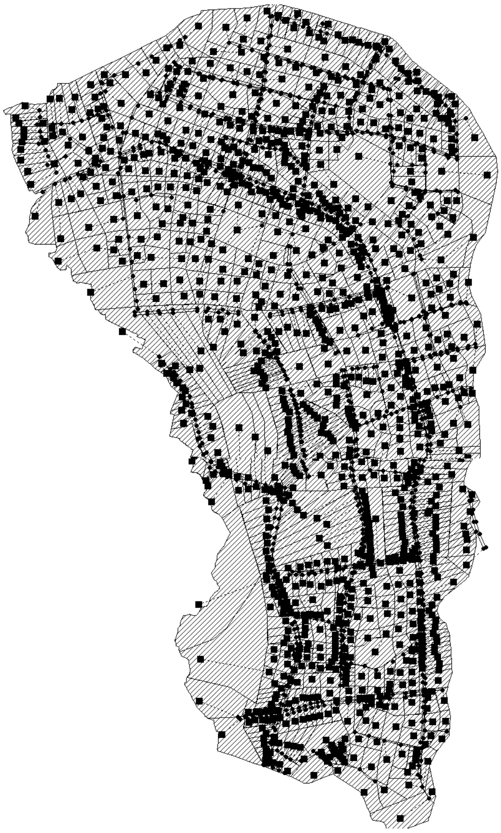

Figure S6. Workspace of research area in SWMM.

(2) Parameter Setting and Calculation

There are two types of parameters in SWMM (Table S1); measurement parameters and calibration parameters. Measurement parameters can be calculated by ArcGIS, while the calibration parameters are the ones within a range of reasonable interval and determined through model calibration.

Table S1. Parameter types of SWMM.

Parameter types	Measurement parameters	Calibration parameters
Catchments	Area, Slope, Impermeability	Width，Roughness coefficient of impermeability, Roughness coefficient of permeability, Surface-depression storage of impermeability, Surface-depression storage of permeability, Zero surface-depression storage of impermeability
Conduits	Shape, Maximum depth, Length	Roughness coefficient
Junctions	The inner bottom elevation, Maximum depth, Flooded area	None
HORTON infiltration models	None	Maximum infiltration rate, Minimum infiltration rate, Decay constant, Drying time

Source: Developed based on the website of SWMM [1] and research of Knighton (2016).

Calculation of Measurement Parameters

[1] https://www.epa.gov/water-research/storm-water-management-model-swmm

Area, length, coordinates X and Y can be calculated using the Calculate Geometry Tool in ArcGIS. Average slope (%) (Figure S7) of the study area can be calculated using the module of Spatial Analyst Tools (Zonal Statistics) of ArcGIS based on DEM.

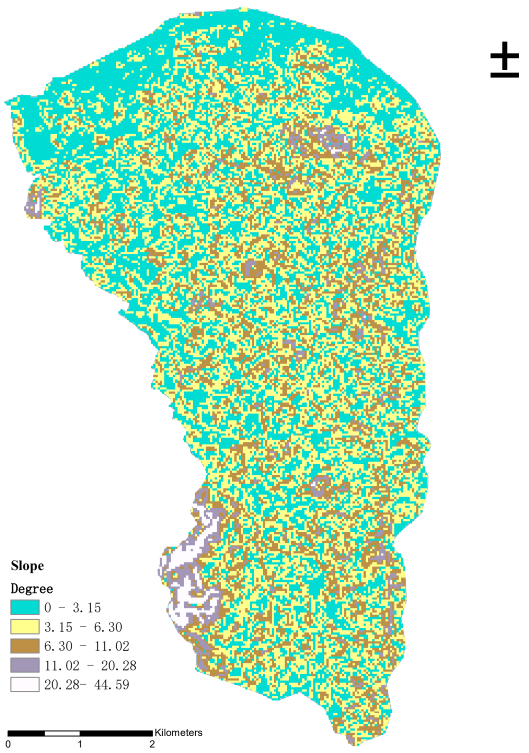

Figure S7. Slope (%) of the research area.

Impermeability depends on the properties of the underlying surfaces of the study area. In this study, the land use map of 2014 is selected and simplified. As shown in Figure S1, there were 11 types of land use was generalized for our research area. The impermeability coefficient is valued based on different land use types (Table S2). The Analyst Tools module (Summary Statistics) of ArcGIS was selected to calculate the impermeability coefficient of different catchments based on the weighted average area of each type of land use.

Table S2. Impermeability coefficients of different land use types.

**Types**	**Permeability**			
Name	Grass, Forest, Farmland, Bare lands, Garden and green lands, Vacant lands, Water areas	Low-density construction lands	High-density construction lands	Transportation lands
Impermeability Coefficient	0	0.8	0.9	1

Source: Cite from Sun (2011).

Calculation of the Calibration Parameters

According to the user manual of the SWMM model [2], the ranges of the different calibration parameters are shown in Tables S3 and S4:

[2] https://nepis.epa.gov/Exe/ZyPDF.cgi?Dockey=P100NYRA.txt

Table S3. Depression storage of different types of underlying surfaces.

Underlying surfaces	Depression storage/mm
Impermeable surface	1.27～2.54
Grass	2.54～5.08
Pasture	5.08
Forest	7.62

Source: Cite from Qin et al. (2013) and Sun (2011).

Table S4. Range of Manning coefficient (N value).

Underlying surfaces	N	Pipe types	N
Smooth-Surfaced Asphalt	0.011	Asbestos-cement pipe	0.011～0.015
Smooth-Surfaced Concrete	0.012	Bricked pipe	0.013～0.017
Conventional Concrete	0.013	Casting ductile iron pipe	0.011～0.015
Wood	0.014	Concrete (entirely)	
Clay	0.015	Smooth- Surfaced	0.012～0.014
Fallow soil（No vegetal residues）	0.05	Unsmooth- Surfaced	0.015～0.017
Cultivated soil（Residual vegetation<20%）	0.06	Corrugated metal pipe	
Cultivated soil（Residual vegetation >20%）	0.17	Flat surface	0.022～0.026
Pasture（Nature）	0.13	Paved inner sole	0.018～0.022
Grass	0.15～0.41	Fiber-Reinforced asphalt lining	0.011～0.015
Forest	0.4～0.8	Plastic conduit (smooth-surfaced)	0.011～0.015
		Concrete pipes	0.011～0.015

Source: Cite from Qin et al. (2013) and Sun (2011).

Considering the true conditions of the study area, such as climate, soil type, underlying surfaces, and pipeline materials, the parameters in this analysis are limited to the ranges in Table S5.

Table S5. Range of calibration parameter of the study area.

Parameter	Value range
Width /m	K*SQRT（area）
N-Impervious	0.010～0.015
N-Pervious	0.10～0.30
Depression storage-Impervious/mm	2～5
Depression storage-Pervious /mm	3～10
Zero-Depression storage-Impervious /%	5～30
Roughness of pipeline	0.013～0.015
Maximum Infiltration Rate/(mm/h)	25.4～254
Minimum Infiltration Rate /(mm/h)	0.3～120.4
Decay Constant	2～7
Drying Time	2～14

Source: Reference to some scholars' research, such as Sun (2011) and Qin et al. (2013).

Among these parameters, "width" plays a dominant role on the accuracy of the model simulation. Width is defined as the ratio between the catchment area and the longest length of the overland flow. In practice, the process of water accumulation and drainage is complicated and the paths of overland flow are difficult to calculate. Therefore, the width cannot be truly measured. The four methods for the indirect measurement of width are listed as follows (Sun, 2011):

a. Width=1.7*MAX (Height, Width);
b. Width=K*Sqrt (Area) (0.2<K<5);
c. Width=K*Perimeter (0<K<1);
d. Width=Area/Flow Length

Among these four methods, b and d are quite commonly used. In this study, all of the four methods have been tested and finally, method b was chosen to calculate the width because the simulation results of b were more accurate.

After building the SWMM model and testing its accuracy, the duration of each urban flooding area can be directly read through the result reports of SWMM.

(3) Drainage capacity performance

Duration is the length of time from the apperance of waterlogging to the fading of waterlogging. It can be determined using the simulation results of SWMM. The precipitation process on 11 May 2014 was used to simulate the urban flooding process, and then the measured data on depth and spatial distribution of urban flooding (on the same day) were used to calibrate the parameters and validate the simulation. The spatial distribution of test points and their values are shown in Figure S8 and Table S6. The simulation results of the depth and spatial distribution of urban floods are shown in Figure S9. There were a total of 12 points, and six of them were applied to calibrate the parameters of SWMM; the other six were used to validate the simulation results (Table S6). The calibration and validation data are shown in Table S7. The existing literature shows that in foreign countries, such as in the city of Cincinnati (Ohio, USA), ±15% was used as the tolerance level of model accuracy for peak depth (Siegrist et al., 2016); while in China, some scholars pointed out that 20% would be acceptable, given the relatively large scale of the simulations (Yu et al., 2012). Considering that this research area is quite large, and that the terrain is complicated, the tolerance level was set equal to or less than 30%. The results demonstrate that the SWMM meets Gongming's most recent modeling standards. The results basically fit the requirements of simulation, but with relative error spread from 1% to 30%. Some of the calibration parameters are shown in Table S8.

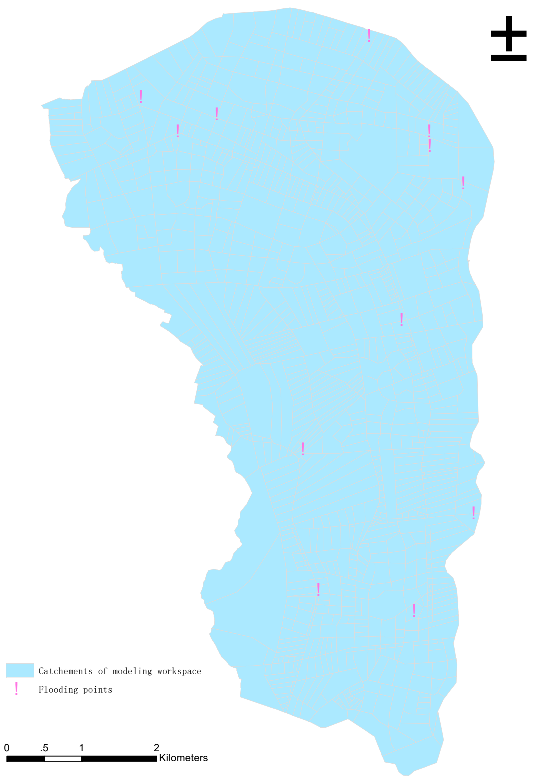

Figure S8. Spatial distribution of the test points of urban flooding in the research area.

Table S6. Measurements of urban flooding points for model testing.

ID	County	Location	Longitude	Latitude	Area	Depth	Flooding Hours
1	Shangcun	Road crossing of Minsheng Street and Xingfa Road	113.903	22.785	1000	0.25	4
2	Shangcun	Road crossing of Nan Ringroad and Bieshu Road	113.907	22.780	2000	0.30	4
3	Shangcun	Road crossing of North Ringroad and Changchun North Road	113.895	22.798	2000	0.30	3
4	Gongming	Road crossing of Huafa North Road and Minsheng Dadao	113.903	22.786	5000	0.20	3
5	Jiangshi	Outside of Yihemoju on Genyu Road	113.887	22.731	3000	0.60	4
6	Jiangshi	Jiangshi Road section of Genyu Road	113.885	22.748	2000	0.50	1
7	Tangwei	Zhoujia Dadao on Songbai Road	113.899	22.764	3000	0.25	3
8	Xiacun	Road crossing of West Ringroad and Keyu Road	113.865	22.791	1000	0.20	4
9	Tianliao	Outside of Weidong Store	113.900	22.729	2000	0.30	3
10	Mashantou	Auto market of Gongming	113.870	22.787	500	0.20	2
11	Jiazitang	Gate of Jiazitang County	113.908	22.740	500	0.25	2

Source: Guangming Wind, Flood, and Drought Prevention Office (2016).

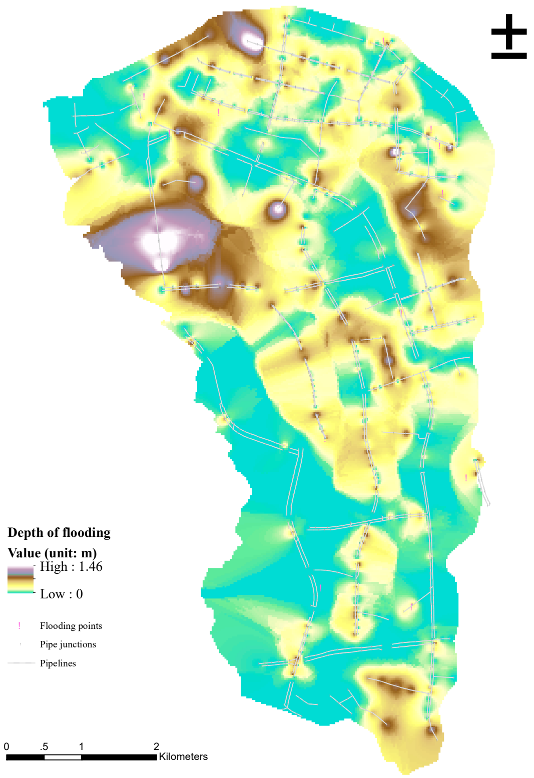

Figure S9. Spatial distribution of depth of urban flooding.

Table S7. Calibration and validation results.

Points ID	Observed (m)	Modeled (m)	Errors (m)	% Difference
		Calibration		
1	0.25	0.255	0.005	2%
2	0.30	0.285	-0.015	-5%
3	0.30	0.320	0.020	6%
4	0.20	0.217	0.017	8%
5	0.20	0.224	0.024	11%
6	0.50	0.453	-0.047	-10%
		Validation		
7	0.50	0.441	-0.059	-13%
8	0.25	0.248	-0.002	-1%
9	0.20	0.174	-0.046	-30%
10	0.30	0.205	-0.085	-41%
11	0.20	0.165	-0.035	-18%
12	0.25	0.226	-0.024	-11%

Table S8. Values of some calibration parameters.

Parameters	Calibration value
K	2
N-Impervious	0.015
N-Pervious	0.15
Depression storage-Impervious/mm	5
Depression storage-Pervious /mm	15
Zero-Depression storage-Impervious /%	25
Roughness	0.013
Maximum Infiltration Rate/(mm/h)	76
Minimum Infiltration Rate /(mm/h)	12
Decay Constant	2
Drying Time	5

The final model was built based on the calibration parameters, and based on the model using the rainfall data of 11 May 2014, the duration could be read from the model. The spatial distribution of the simulated duration results is shown in Figure S10. It should be mentioned that the parameter 'duration' is different from that called 'flooding hours' shown in Table S9. The parameter 'Flooding hours', shows the number of hours at peak depth, while 'duration' is the length of time from the apperance of waterlogging to the fading of waterlogging, which can be read directly from the simulation. Based on the simulated duration results, we were able to find that for most of the places that appeared waterlogged on that day in the resarch area; the duration was from 0 to 21.83 hours. We found that there were mainly two long duration belts in this area: one in the northern part of the research area, and the other in the middle of the research area, in a densely populated urban area.

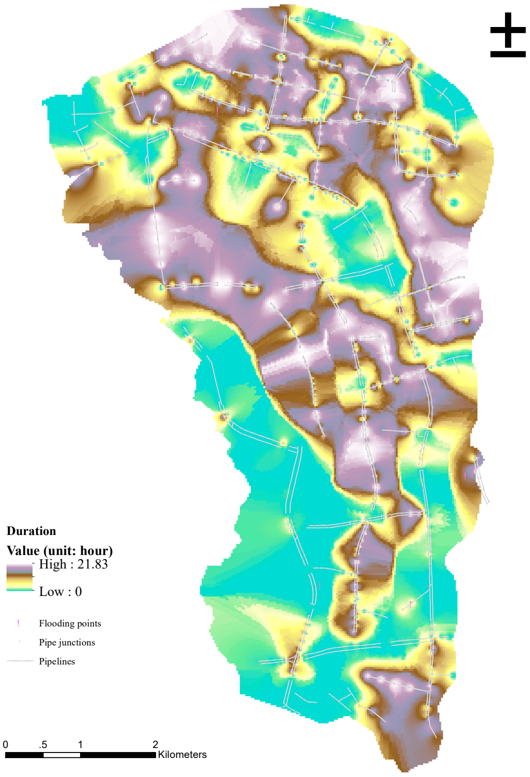

Figure S10. Spatial distribution of drainage capacity.

Duration of urban flooding can reflect the drainage capacity of urban drainage systems, with longer duration indicating low drainage capacity. To dichotomize duration, it was equally divided into four continuous arrays based on original data, and a score was assigned to each array, and then drainage capacity could be scored (Table S9). The drainage capacity was spatially linked with the living places of the residents that answered the questionnaire. The spatial distribution of the domiciles of these residents was identified using the names of their residence zones. Then, making use of the spatial analyst tool ArcGIS, the drainage capacities were extracted to the geometric central points of the residence zones. Finally, the drainage capacity in the area nearest each resident (according to their residence zones) was generated.

Table S9. Scoring for drainage capacity.

Duration (hours)	0-4.33	4.33-8.66	8.66-13.00	13.00-17.33	17.33-21.66
Score of drainage capacity	10.00	7.50	5.00	2.50	0.00

Reference

Knighton, J., Lennon, E., Bastidas, L., and White, E. (2016). Stormwater detention system parameter sensitivity and uncertainty analysis using SWMM. *Journal of Hydrologic Engineering, 21*(8), 05016014.

Qin, H. P., Li, Z. X., and Fu, G. (2013). The effects of low impact development on urban flooding under different rainfall characteristics. *Journal of environmental management, 129*, 577-585.

Sun, A. L. (2011). Risk assessment of rainstorm waterlogging based on scenario simulation. *Shanghai: East China Normal University.*
